# 1-(4-Fluoro­phen­yl)-2-(1*H*-1,2,4-triazol-1-yl)ethanone hemihydrate

**DOI:** 10.1107/S1600536811045429

**Published:** 2011-11-05

**Authors:** Dong-liang Liu, Chen Li, Xin Tian, Song Li, Tao Xiao

**Affiliations:** a Department of Applied Chemistry, College of Science, Nanjing University of Technology, Nanjing 210009, People’s Republic of China

## Abstract

In the title compound, C_10_H_8_FN_3_O·0.5H_2_O, the dihedral angle between the mean planes of the rings is 99.80 (4)°. The water mol­ecule lies on a twofold axis. Weak inter­molecular O—H⋯N and C—H⋯O hydrogen bonds link one water mol­ecule with four phenyl­ethanone mol­ecules, while inter­molecular C—H⋯O hydrogen bonds involving the ketone group link phenyl­ethanone mol­ecules into layers parallel to (100).

## Related literature

For related compounds containing a 2-(1*H*-1,2,4-triazol-1-yl)-1-phenyl­ethanone fragment, see: Akira *et al.* (1985 ▶); Yoshimi *et al.* (2000 ▶); Yuan *et al.* (2007 ▶); Tao *et al.* (2007 ▶). For standard bond lengths, see: Allen *et al.* (1987 ▶).
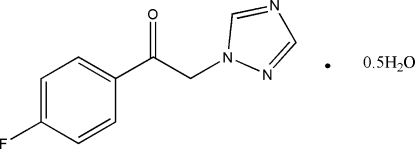


## Experimental

### 

#### Crystal data

C_10_H_8_FN_3_O·0.5H_2_O
*M* *_r_* = 214.2
Orthorhombic, 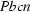

*a* = 24.419 (5) Å
*b* = 10.147 (2) Å
*c* = 8.2410 (16) Å
*V* = 2042.0 (7) Å^3^
*Z* = 8
Mo *K*α radiation
μ = 0.11 mm^−1^
*T* = 293 K
0.3 × 0.2 × 0.2 mm

#### Data collection

Enraf–Nonius CAD-4 diffractometer
Absorption correction: ψ scan (North *et al.*, 1968 ▶) *T*_min_ = 0.968, *T*_max_ = 0.978
3626 measured reflections
1844 independent reflections
1087 reflections with *I* > 2σ(*I*)
*R*_int_ = 0.057
3 standard reflections every 200 reflections  intensity decay: 1%

#### Refinement

*R*[*F* ^2^ > 2σ(*F* ^2^)] = 0.054
*wR*(*F* ^2^) = 0.160
*S* = 1.00
1844 reflections
145 parameters
H atoms treated by a mixture of independent and constrained refinement
Δρ_max_ = 0.17 e Å^−3^
Δρ_min_ = −0.15 e Å^−3^

### 

Data collection: *CAD-4 EXPRESS* (Enraf–Nonius, 1994 ▶); cell refinement: *CAD-4 EXPRESS*; data reduction: *XCAD4* (Harms & Wocadlo, 1995 ▶); program(s) used to solve structure: *SHELXS97* (Sheldrick, 2008 ▶); program(s) used to refine structure: *SHELXL97* (Sheldrick, 2008 ▶); molecular graphics: *PLATON* (Spek, 2009 ▶); software used to prepare material for publication: *SHELXTL* (Sheldrick, 2008 ▶).

## Supplementary Material

Crystal structure: contains datablock(s) I, global. DOI: 10.1107/S1600536811045429/zq2131sup1.cif

Structure factors: contains datablock(s) I. DOI: 10.1107/S1600536811045429/zq2131Isup2.hkl

Supplementary material file. DOI: 10.1107/S1600536811045429/zq2131Isup3.cml

Additional supplementary materials:  crystallographic information; 3D view; checkCIF report

## Figures and Tables

**Table 1:** Hydrogen-bond geometry (Å, °)

*D*—H⋯*A*	*D*—H	H⋯*A*	*D*⋯*A*	*D*—H⋯*A*
O*W*—H*WA*⋯N1	0.80 (4)	2.18 (4)	2.957 (3)	166 (4)
C2—H2*B*⋯O^i^	0.93	2.54	3.309 (3)	140
C3—H3*B*⋯O^ii^	0.97	2.51	3.459 (3)	166
C7—H7*A*⋯O*W*^iii^	0.93	2.49	3.407 (4)	169

Symmetry codes: (i) 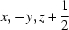; (ii) 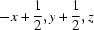; (iii) 
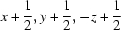
.

**Fig. 1.**
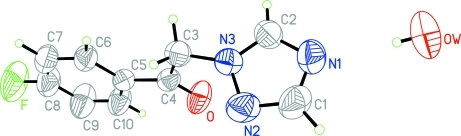
The molecular structure of the title compound with the atom-numbering scheme. Displacement ellipsoids are drawn at the 50% probability level.

**Fig. 2.**
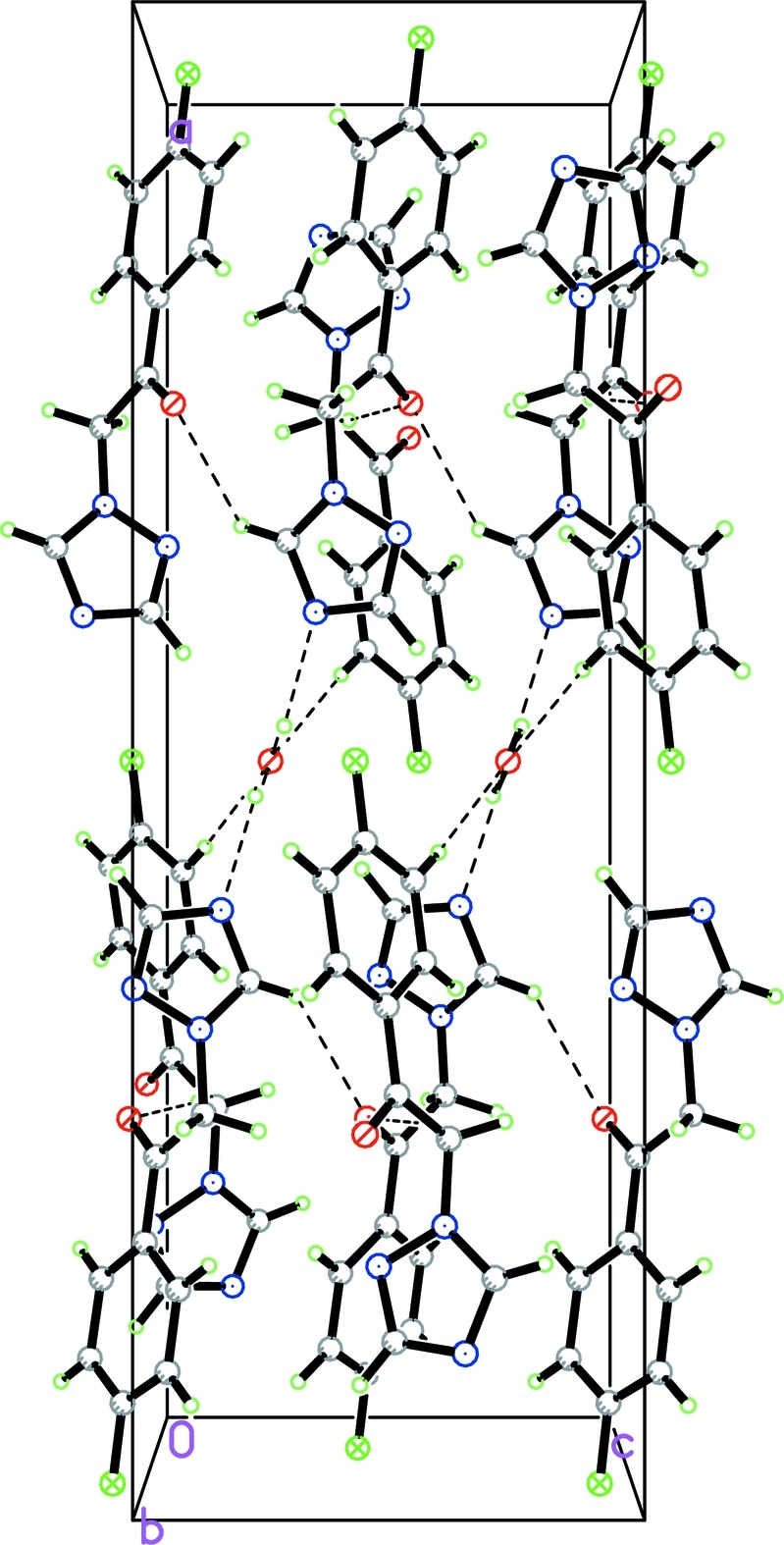
A packing diagram of the title compound showing the intermolecular O—H···N hydrogen bonds (dashed lines).

## supplementary crystallographic information

### Comment

The title compound is the key intermediate in the synthesis of a new kind of antifungal drug (Yoshimi *et al.*, 2000; Akira *et al.*, 1985). The crystal structure determination has been carried out in order to elucidate the molecular conformation.

The molecular structure of the title compound is reported in Fig. 1. The bond lengths (Allen *et al.*, 1987) and angles are within normal ranges. The dihedral angle between the mean planes of the N1/N2/C9–C11 and C1–C6 rings is 99.80 (4)°. The solvent molecules of water lie on two-fold axes. The second position of the hydrogen atom is reproduced by the symmetry operation (-x, y, 0.5-z). Weak intermolecular O—H···N (Fig. 2) and C—H···O hydrogen bonds link one isolated water molecule with four phenylethanone molecules while intermolecular C—H···O hydrogen bonds involving the ketone group link phenylethanone molecules together into layers parallel to (100).

### Experimental

Sodium hydride (4.8 g, 120 mmol) was suspended in dimethylformamide (DMF, 30 ml). Triazole (8.28 g, 120 mmol) dissolved in DMF (30 ml) was slowly added dropwise at 273 K, and reacted at room temperature for 30 min. 2-Chloro-1-(4-fluorophenyl)ethanone (15.48 g, 90 mmol) dissolved in DMF (30 ml) was then slowly added dropwise, and reacted at room temperature for 4 h. The mixture was placed in ice-water (300 ml), and 1 mol hydrochloric acid (50 ml) was then added. After filtration, the filtrate was neutralized with sodium bicarbonate to pH = 6, and a yellow deposit was obtained. Recrystallization with ethanol yielded a white deposit (m.p. 397–400 K). Crystals suitable for a X-ray analysis study were obtained by dissolving the crude product (1.0 g) in 95% ethanol (30 ml) and then allowing the solution to evaporate slowly at room temperature for about 7 days.

### Refinement

The water H atom (the second position being obtained by a symmetry operation) was located in a Fourier difference map and freely refined with *U*_iso_(H) = 1.2*U*_eq_(O). The other H atoms were positioned geometrically with C—H = 0.93 Å (aromatic) and 0.97 Å (methylene) and constrained to ride on their parent atoms with *U*_iso_(H) = 1.2*U*_eq_(C).

### Crystal data

**Table:**

C_10_H_8_FN_3_O·0.5H_2_O	*D*_x_ = 1.394 Mg m^−^^3^
*M**_r_* = 214.2	Melting point: 397 K
Orthorhombic, *P**b**c**n*	Mo *K*α radiation, λ = 0.71073 Å
Hall symbol: -P 2n 2ab	Cell parameters from 25 reflections
*a* = 24.419 (5) Å	θ = 9–13°
*b* = 10.147 (2) Å	µ = 0.11 mm^−^^1^
*c* = 8.2410 (16) Å	*T* = 293 K
*V* = 2042.0 (7) Å^3^	Prism, yellow
*Z* = 8	0.3 × 0.2 × 0.2 mm
*F*(000) = 888	

### Data collection

**Table:**

Enraf–Nonius CAD-4 diffractometer	1087 reflections with *I* > 2σ(*I*)
Radiation source: fine-focus sealed tube	*R*_int_ = 0.057
graphite	θ_max_ = 25.4°, θ_min_ = 1.7°
ω/2θ scans	*h* = −29→29
Absorption correction: ψ scan (North *et al.*, 1968)	*k* = −12→0
*T*_min_ = 0.968, *T*_max_ = 0.978	*l* = 0→9
3626 measured reflections	3 standard reflections every 200 reflections
1844 independent reflections	intensity decay: 1%

### Refinement

**Table:**

Refinement on *F*^2^	Secondary atom site location: difference Fourier map
Least-squares matrix: full	Hydrogen site location: inferred from neighbouring sites
*R*[*F*^2^ > 2σ(*F*^2^)] = 0.054	H atoms treated by a mixture of independent and constrained refinement
*wR*(*F*^2^) = 0.160	*w* = 1/[σ^2^(*F*_o_^2^) + (0.084*P*)^2^] where *P* = (*F*_o_^2^ + 2*F*_c_^2^)/3
*S* = 1.00	(Δ/σ)_max_ < 0.001
1844 reflections	Δρ_max_ = 0.17 e Å^−^^3^
145 parameters	Δρ_min_ = −0.15 e Å^−^^3^
0 restraints	Extinction correction: *SHELXL97* (Sheldrick, 2008), Fc^*^=kFc[1+0.001xFc^2^λ^3^/sin(2θ)]^-1/4^
Primary atom site location: structure-invariant direct methods	Extinction coefficient: 0.062 (7)

### Special details

**Table:**

Geometry. All e.s.d.'s (except the e.s.d. in the dihedral angle between two l.s. planes) are estimated using the full covariance matrix. The cell e.s.d.'s are taken into account individually in the estimation of e.s.d.'s in distances, angles and torsion angles; correlations between e.s.d.'s in cell parameters are only used when they are defined by crystal symmetry. An approximate (isotropic) treatment of cell e.s.d.'s is used for estimating e.s.d.'s involving l.s. planes.
Refinement. Refinement of *F*^2^ against ALL reflections. The weighted *R*-factor *wR* and goodness of fit *S* are based on *F*^2^, conventional *R*-factors *R* are based on *F*, with *F* set to zero for negative *F*^2^. The threshold expression of *F*^2^ > σ(*F*^2^) is used only for calculating *R*-factors(gt) *etc*. and is not relevant to the choice of reflections for refinement. *R*-factors based on *F*^2^ are statistically about twice as large as those based on *F*, and *R*- factors based on ALL data will be even larger.

### Fractional atomic coordinates and isotropic or equivalent isotropic displacement parameters (Å^2^)

**Table:**

	*x*	*y*	*z*	*U*_iso_*/*U*_eq_	
O	0.25394 (7)	0.00400 (18)	−0.0459 (2)	0.0749 (6)	
F	0.50013 (8)	0.1731 (3)	−0.0667 (3)	0.1359 (10)	
N1	0.10453 (9)	0.0848 (3)	0.1516 (3)	0.0769 (8)	
C1	0.10833 (13)	0.1521 (3)	0.0121 (4)	0.0774 (9)	
H1B	0.0787	0.1626	−0.0575	0.093*	
N2	0.15669 (10)	0.2021 (3)	−0.0199 (3)	0.0722 (7)	
C2	0.15429 (11)	0.0941 (3)	0.2103 (3)	0.0640 (7)	
H2B	0.1658	0.0569	0.3076	0.077*	
N3	0.18595 (8)	0.16374 (19)	0.1122 (2)	0.0546 (6)	
C3	0.24381 (10)	0.1914 (2)	0.1215 (3)	0.0539 (7)	
H3A	0.2558	0.1837	0.2333	0.065*	
H3B	0.2504	0.2812	0.0864	0.065*	
C4	0.27685 (10)	0.0978 (2)	0.0168 (3)	0.0492 (6)	
C5	0.33581 (10)	0.1223 (2)	−0.0041 (3)	0.0496 (7)	
C6	0.36302 (11)	0.2249 (3)	0.0712 (3)	0.0608 (7)	
H6A	0.3436	0.2831	0.1367	0.073*	
C7	0.41853 (13)	0.2421 (3)	0.0504 (4)	0.0759 (9)	
H7A	0.4370	0.3103	0.1024	0.091*	
C8	0.44541 (13)	0.1573 (4)	−0.0475 (4)	0.0867 (10)	
C9	0.42071 (14)	0.0552 (3)	−0.1240 (4)	0.1019 (12)	
H9A	0.4407	−0.0014	−0.1901	0.122*	
C10	0.36552 (12)	0.0370 (3)	−0.1017 (3)	0.0778 (9)	
H10A	0.3479	−0.0330	−0.1525	0.093*	
OW	0.0000	−0.0387 (3)	0.2500	0.1023 (13)	
HWA	0.0248 (15)	0.006 (4)	0.220 (5)	0.123*	

### Atomic displacement parameters (Å^2^)

**Table:**

	*U*^11^	*U*^22^	*U*^33^	*U*^12^	*U*^13^	*U*^23^
O	0.0593 (11)	0.0603 (11)	0.1051 (14)	−0.0056 (10)	−0.0064 (11)	−0.0288 (11)
F	0.0577 (12)	0.172 (2)	0.178 (2)	−0.0314 (14)	0.0201 (13)	−0.0477 (18)
N1	0.0560 (16)	0.0907 (18)	0.0842 (17)	0.0002 (13)	0.0055 (13)	−0.0031 (15)
C1	0.0541 (19)	0.099 (2)	0.079 (2)	0.0195 (17)	−0.0088 (15)	−0.0097 (19)
N2	0.0639 (16)	0.0880 (17)	0.0647 (15)	0.0182 (13)	−0.0064 (12)	0.0102 (12)
C2	0.0618 (18)	0.0686 (17)	0.0616 (16)	0.0001 (14)	0.0035 (14)	0.0044 (14)
N3	0.0553 (13)	0.0557 (12)	0.0528 (12)	0.0055 (10)	−0.0002 (10)	0.0006 (10)
C3	0.0585 (16)	0.0502 (13)	0.0530 (13)	−0.0014 (11)	0.0003 (12)	−0.0006 (12)
C4	0.0551 (15)	0.0431 (12)	0.0495 (14)	−0.0019 (11)	−0.0063 (11)	0.0005 (11)
C5	0.0520 (15)	0.0504 (14)	0.0465 (13)	−0.0037 (11)	−0.0024 (11)	0.0004 (11)
C6	0.0620 (17)	0.0580 (15)	0.0623 (15)	−0.0105 (13)	0.0025 (13)	−0.0093 (13)
C7	0.069 (2)	0.080 (2)	0.0786 (19)	−0.0229 (16)	0.0005 (16)	−0.0123 (16)
C8	0.057 (2)	0.107 (3)	0.096 (2)	−0.0199 (18)	0.0096 (17)	−0.015 (2)
C9	0.066 (2)	0.113 (3)	0.126 (3)	−0.0109 (19)	0.027 (2)	−0.053 (2)
C10	0.0627 (19)	0.082 (2)	0.088 (2)	−0.0122 (15)	0.0084 (16)	−0.0342 (17)
OW	0.071 (2)	0.085 (2)	0.150 (3)	0.000	0.034 (2)	0.000

### Geometric parameters (Å, °)

**Table:**

O—C4	1.219 (3)	C4—C5	1.471 (3)
F—C8	1.355 (3)	C5—C6	1.382 (3)
N1—C2	1.311 (3)	C5—C10	1.387 (3)
N1—C1	1.341 (4)	C6—C7	1.377 (4)
C1—N2	1.312 (4)	C6—H6A	0.9300
C1—H1B	0.9300	C7—C8	1.350 (4)
N2—N3	1.359 (3)	C7—H7A	0.9300
C2—N3	1.323 (3)	C8—C9	1.354 (4)
C2—H2B	0.9300	C9—C10	1.372 (4)
N3—C3	1.443 (3)	C9—H9A	0.9300
C3—C4	1.516 (3)	C10—H10A	0.9300
C3—H3A	0.9700	OW—HWA	0.79 (4)
C3—H3B	0.9700		
			
C2—N1—C1	102.4 (2)	C5—C4—C3	118.8 (2)
N2—C1—N1	115.6 (3)	C6—C5—C10	118.6 (2)
N2—C1—H1B	122.2	C6—C5—C4	123.1 (2)
N1—C1—H1B	122.2	C10—C5—C4	118.3 (2)
C1—N2—N3	101.6 (2)	C7—C6—C5	120.9 (3)
N1—C2—N3	110.8 (2)	C7—C6—H6A	119.6
N1—C2—H2B	124.6	C5—C6—H6A	119.6
N3—C2—H2B	124.6	C8—C7—C6	118.1 (3)
C2—N3—N2	109.6 (2)	C8—C7—H7A	120.9
C2—N3—C3	130.1 (2)	C6—C7—H7A	120.9
N2—N3—C3	120.1 (2)	C7—C8—C9	123.3 (3)
N3—C3—C4	111.66 (19)	C7—C8—F	118.3 (3)
N3—C3—H3A	109.3	C9—C8—F	118.4 (3)
C4—C3—H3A	109.3	C8—C9—C10	118.6 (3)
N3—C3—H3B	109.3	C8—C9—H9A	120.7
C4—C3—H3B	109.3	C10—C9—H9A	120.7
H3A—C3—H3B	107.9	C9—C10—C5	120.5 (3)
O—C4—C5	122.1 (2)	C9—C10—H10A	119.8
O—C4—C3	119.1 (2)	C5—C10—H10A	119.8
			
C2—N1—C1—N2	0.1 (3)	O—C4—C5—C10	1.6 (4)
N1—C1—N2—N3	−0.5 (3)	C3—C4—C5—C10	−179.2 (2)
C1—N1—C2—N3	0.4 (3)	C10—C5—C6—C7	−0.2 (4)
N1—C2—N3—N2	−0.8 (3)	C4—C5—C6—C7	178.6 (2)
N1—C2—N3—C3	−175.2 (2)	C5—C6—C7—C8	1.0 (4)
C1—N2—N3—C2	0.7 (3)	C6—C7—C8—C9	−1.1 (5)
C1—N2—N3—C3	175.8 (2)	C6—C7—C8—F	−179.2 (3)
C2—N3—C3—C4	97.0 (3)	C7—C8—C9—C10	0.3 (6)
N2—N3—C3—C4	−76.9 (3)	F—C8—C9—C10	178.5 (3)
N3—C3—C4—O	−8.4 (3)	C8—C9—C10—C5	0.5 (5)
N3—C3—C4—C5	172.43 (19)	C6—C5—C10—C9	−0.6 (4)
O—C4—C5—C6	−177.3 (2)	C4—C5—C10—C9	−179.5 (3)
C3—C4—C5—C6	1.9 (3)		

### Hydrogen-bond geometry (Å, °)

**Table:**

*D*—H···*A*	*D*—H	H···*A*	*D*···*A*	*D*—H···*A*
OW—HWA···N1	0.80 (4)	2.18 (4)	2.957 (3)	166 (4)
C2—H2B···O^i^	0.93	2.54	3.309 (3)	140.
C3—H3B···O^ii^	0.97	2.51	3.459 (3)	166.
C7—H7A···OW^iii^	0.93	2.49	3.407 (4)	169.

Symmetry codes: (i) *x*, −*y*, *z*+1/2; (ii) −*x*+1/2, *y*+1/2, *z*; (iii) *x*+1/2, *y*+1/2, −*z*+1/2.
